# Hemodynamic response to familiar faces in children with ADHD

**DOI:** 10.1186/s13030-019-0172-1

**Published:** 2019-11-28

**Authors:** Keiichi Shimamura, Takeshi Inoue, Hiroko Ichikawa, Emi Nakato, Yuiko Sakuta, So Kanazawa, Masami K. Yamaguchi, Ryusuke Kakigi, Ryoichi Sakuta

**Affiliations:** 1 Child Development and Psychosomatic Medicine Center, Dokkyo Medical University Saitama Medical Center, 2-1-50, Minami-Koshigaya, Koshigaya-shi, Saitama-Ken, 343-8555 Japan; 2 Department of Pediatrics, Dokkyo Medical University Saitama Medical Center, Saitama, Japan; 3 Department of Diagnostic Imaging, Program in Neurosciences & Mental Health, Hospital for Sick Children, Toronto, Ontario Canada; 4 Faculty of Science and Technology, Tokyo University of Science, Chiba, Japan; 5 Department of Clothing, Osaka Shoin Women’s University, Osaka, Japan; 6 Faculty of Human Life Sciences, Jissen Women’s University, Tokyo, Japan; 7 Department of Psychology, Japan Women’s University, Kanagawa, Japan; 8 Department of Psychology, Chuo University, Tokyo, Japan; 9 Department of Integrative Physiology, National Institute for Physiological Sciences, Aichi, Japan

**Keywords:** ADHD, Face recognition, Familiar face, fNIRS, The superior temporal sulci

## Abstract

**Background:**

School-age children with attention deficit hyperactivity disorder (ADHD) have difficulties in interpersonal relationships, in addition to impaired facial expression perception and recognition. For successful social interactions, the ability to discriminate between familiar and unfamiliar faces is critical. However, there are no published reports on the recognition of familiar and unfamiliar faces by children with ADHD.

**Methods:**

We evaluated the neural correlates of familiar and unfamiliar facial recognition in children with ADHD compared to typically developing (TD) children. We used functional near-infrared spectroscopy (fNIRS) to measure hemodynamic responses on the bilateral temporal regions while participants looked at photographs of familiar and unfamiliar faces. Nine boys with ADHD and 14 age-matched TD boys participated in the study. fNIRS data were Z-scored prior to analysis.

**Results:**

During familiar face processing, TD children only showed significant activity in the late phase, while ADHD children showed significant activity in both the early and late phases. Additionally, the boys with ADHD did not show right hemispheric lateralization to familiar faces.

**Conclusions:**

This study is the first to assess brain activity during familiar face processing in boys with ADHD using fNIRS. These findings of atypical patterns of brain activity in boys with ADHD may be related to social cognitive impairments from ADHD.

## Background

Attention deficit hyperactivity disorder (ADHD) is characterized by major symptoms of hyperactivity, impulsivity, and inattention, which result in problems in the individual’s quality of life [1, 2]. It is believed that it is due to these symptoms that school-age children with ADHD have difficulties in interpersonal relationships [3]; however, children with ADHD may have additional social cognitive impairments [4]. These impairments related to ADHD frequently lead to difficulties in daily life and may occasionally result in stressors. Understanding the mechanisms of these impairments and stressors is crucial in developing supports for children with ADHD that prevent secondary disabilities such as school refusal and psychosomatic disorders.

The early development of facial processing has been considered fundamental to the development of social abilities and theory of mind [5]. Several abilities compose social cognition, including the ability to perceive the emotions of another person from affective prosody, faces, and body posture [4]. It has been shown repeatedly that children with ADHD, particularly those that are school-age, have impaired facial expression perception and recognition [4, 6, 7].

However, there is limited knowledge regarding basic facial processing in ADHD. Tye et al. (2013) were the first to explore the face inversion effects and gaze processing in children with ADHD using event related potentials (ERPs) [8]. They found that children with ADHD displayed a reduced face inversion effect on P1 latency compared with TD children. Sinzig et al. (2008) reported that the ability of facial affect recognition is reduced in children suffering from ADHD symptoms, both in autistic and ADHD children [9]. Several lines of evidence have suggested differential processing between familiar and unfamiliar faces with behavioral as well as neural findings in typical adult studies [10, 11]. Therefore, we hypothesized that ADHD children, who present with social cognitive impairments, may have different familiar and unfamiliar facial recognition compared with TD children.

fNIRS is a neuroimaging device which provides an index of brain function by measuring changes in the concentration of oxygenated hemoglobin (oxy-Hb), deoxygenated hemoglobin (deoxy-Hb), and total-hemoglobin (total-Hb) using observation of the diffuse transmittance of near-infrared light at an appropriate range of wavelengths [12, 13]. fNIRS has been utilized for more than a decade to examine brain activity during various cognitive tasks in children with neurodevelopmental disorders [7, 14–19]. Compared to other neuroimaging devices such as functional magnetic resonance imaging (fMRI), fNIRS has clear advantages, including that it is a completely silent process, has a high time.

resolution, and provides a non-intrusive environment requiring less restriction of the body and head [7, 20]. fNIRS has been adopted in studies examining the brain activity of children with ADHD while they perform cognitive tasks [16–19, 21]. These studies measured the hemodynamic response in the prefrontal areas, which play an important role in processing executive cognitive functions. With regard to facial recognition, fNIRS also has been utilized to measure neural activities in the temporal area, including the superior temporal sulci (STS), which is known to play an important role in processing faces [22]; this is consistent with other hemodynamic evidence of facial processing in the temporal cortex of the brain [7, 20, 23–27].

To our knowledge, there are no published reports on the recognition of familiar and unfamiliar faces in children with ADHD. Thus, the purpose of this study is to compare the neural correlates involved in familiar (mother) and unfamiliar facial recognition in children with and without ADHD using a fNIRS system, which was placed over the bilateral temporal regions.

## Materials and methods

### Participants

Seventeen ADHD boys and 16 TD boys were recruited. All diagnoses were guided by the DSM-5 [2] and were made by two pediatric neurologists who worked with neurodevelopmental disorders for over 5 years. Exclusion criteria included learning, language, neurological or developmental disabilities. Current study protocols were approved by the Ethical Committee of the Dokkyo Medical University Saitama Medical Center (hosp-k 24,016). We obtained written informed consent from the participants and their parents. The experiments were conducted according to the Declaration of Helsinki.

After excluding subjects whose fNIRS data failed the motion criteria (frequent sharp changes in the time series of the raw fNIRS data), 23 subjects remained: 9 boys with ADHD (mean age, 9 years 9 months; SD, 1 year 6 months; range, 8–13 years), and 14 TD boys (mean age, 9 years 4 months; SD, 1 year 6 months; range, 7–12 years) (Table 1). All participants were right-handed. Six out of nine boys with ADHD received methylphenidate, and one ADHD boy received atomoxetine. All of them participated in the examination for 4–6 h following medication. ADHD and TD subjects were not different according to age (see Table 1). The ADHD-Rating Scale scores were 34.2 (range, 11–52; SD, 13.9) in the ADHD boys and 9.8 (range, 2–18; SD, 4.5) in the TD boys. The Wechsler Intelligence Scale of Children Third Edition (WISC-III) full IQ scores of the TD boys (mean, 111.1; SD, 10.0; range, 90–129) were significantly higher (*t* = 3.68, *p* = 0.048) than those of the ADHD participants (mean, 94.1; SD, 16.2; range, 78–125).

**Table 1:** Demographic and clinical characteristics of ADHD and Typical boys. The participants were 23 Japanese boys aged 7–13 years: 9 boys with ADHD and 14 TD boys. Fourteen TD boys were matched with the ADHD subjects according to age. The participants were limited to boys

ADHD				Typical			
ID	age	Full IQ	ADHD-RS	ID	age	Full IQ	ADHD-RS
1	8y2m	106	41	10	7y3m	124	13
2	8y4m	91	38	11	7y9m	110	12
3	8y10m	82	52	12	7y9m	129	13
4	9y0m	110	11	13	8y0m	106	5
5	9y7m	125	15	14	8y5m	115	10
6	10y0m	78	29	15	8y7m	90	12
7	10y0m	84	48	16	8y8m	107	13
8	10y6m	79	34	17	8y9m	124	3
9	13y4m	92	40	18	10y1m	113	7
				19	10y4m	105	10
				20	10y5m	102	18
				21	10y11m	110	12
				22	11y0m	107	7
				23	12y6m	113	2
range	8y-13y	78-125	11-52	range	7y-12y	90-129	2-18
mean	9y9m	94.1	34.2	mean	9y4m	111.1	9.8
SD	1y6m	16.2	13.9	SD	1y6m	10.0	4.5

### Stimuli and design

**Fig. 1:**
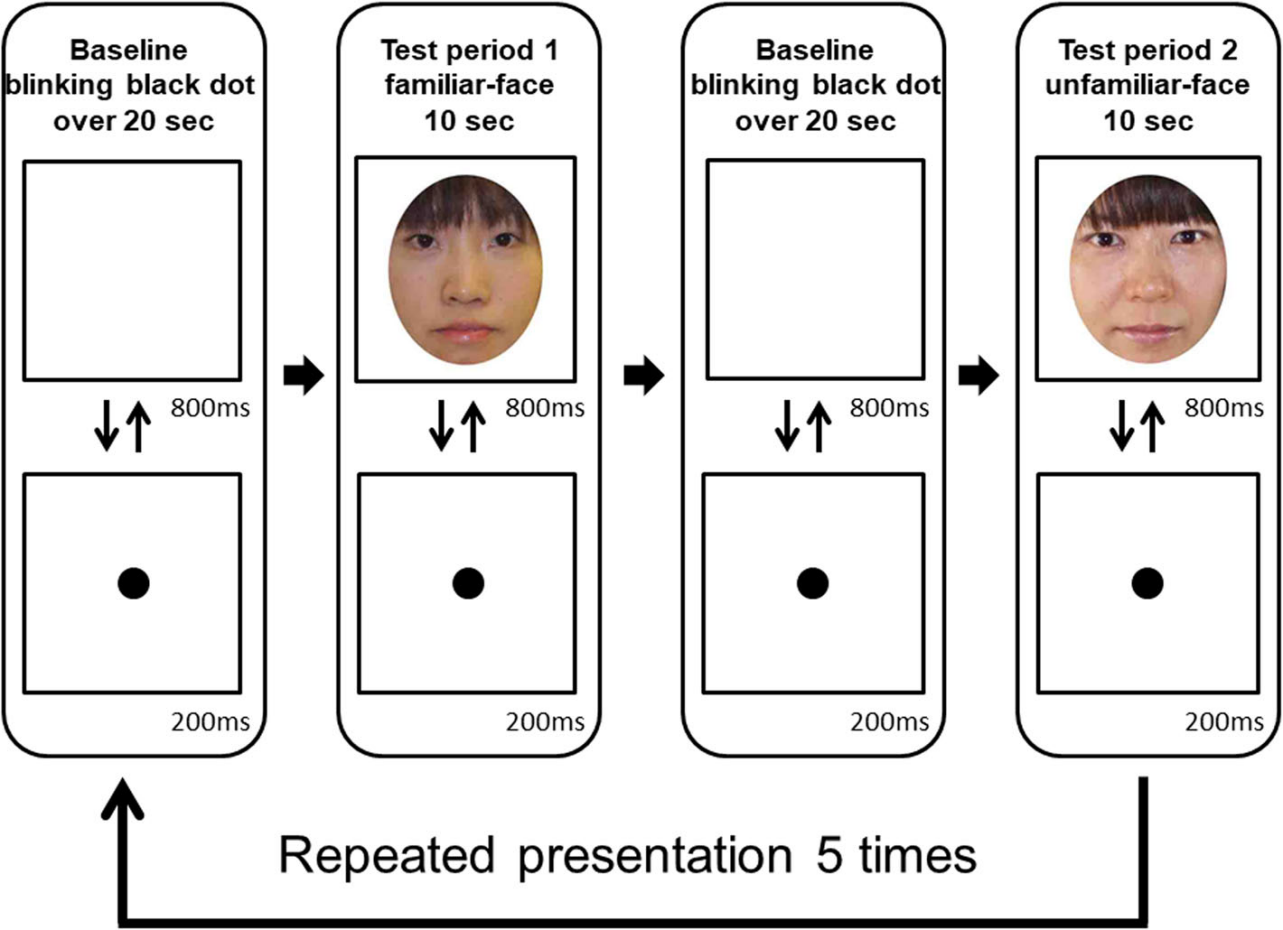
Experimental procedure. Experimental procedure. In each trial, the baseline phase consisted of images of a black dot with a duration over 20 s. The test phase consisted of the familiar face and the unfamiliar face. The duration of the test phase was 10 s. The presentation order of test phase 1 and 2 was changed alternately for each boy

Each child was examined while sitting on a chair and facing a computer screen approximately 50 cm away. The boys watched the stimuli passively while their hemodynamic response was measured, and their behavior was recorded on video during the test. The sequence of stimulus presentations was composed of baseline and test periods. The test periods consisted of two stimulus conditions: familiar face and unfamiliar face conditions. In those conditions, the participants looked at familiar face or unfamiliar face color photographs, respectively (Fig. 1). The order of the conditions was counterbalanced across the participants. The familiar face photograph was the participant’s mother’s face. The unfamiliar face photographs were the faces of age-matched unknown females. To exclude the external features of the faces, we removed the hair from the face images so that only the internal features were visible. The sizes of the photographs were approximately 13 × 10 deg for the faces. The duration of the test period was fixed at 10 s. Each face image was shown 10 times in one test period. The duration of each face image was 800 ms, and the 200 ms inter-stimulus interval was filled by the presentation of a fixation point (a blinking black dot, 3.5 × 3.5 deg). Each test period was preceded by a baseline period that was at least 20 s long. During the baseline period, the screen of the monitor was a uniform blank white screen. The blank screen was presented for 800 ms, and a 200 ms inter-blank screen interval was filled with the same fixation point (a blinking black dot), the same as in the test period. The results obtained from viewing the blank screen were used as the baseline. To draw and retain the attention of the boys, both face stimuli and the blank screen were accompanied by a beeping sound, as in previous studies [26].

### Recording

We used a Hitachi ETG-4000 system (Hitachi Medical, Chiba, Japan), which can record 24 channels simultaneously with 12 channels over the right temporal area and 12 over the left. This instrument generates two wavelengths of NIR (695 nm and 830 nm) and measures the time courses of oxyhemoglobin (oxy-Hb) and deoxyhemoglobin (deoxy-Hb) levels and their sum (total hemoglobin: total-Hb) with a 0.1 s time resolution. The fNIRS probes contained nine optical fibers (3 × 3 arrays) composed of five emitters and four detectors. The distance between the emitters and the detectors was set at 3 cm. Each pair of adjacent emitting and detecting fibers defined a single measurement channel.

**Fig. 2:**
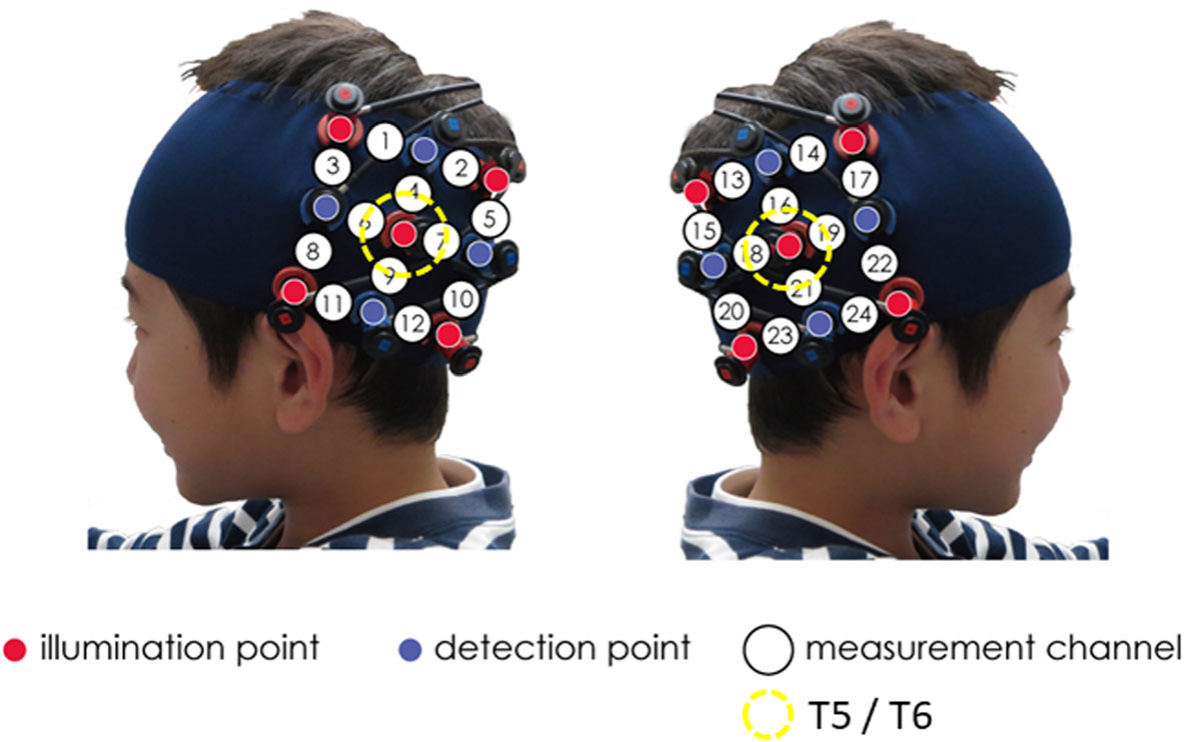
Placement of NIRS probes. The placement of NIRS probes. The probes were placed over the left and right temporal areas centering at T5 and T6 of the International 10–20 system. T5/T6 is in the yellow circle. The distance between the probes was set at 3 cm

The placement of the probes was set on the bilateral temporal cortices centered at T5 and T6 according to the International 10–20 system [28] (Fig. 2). This location of the probes was the same as that reported by Otsuka et al. [24]. When the probes were positioned, the experimenter checked to see if the fibers were touching the boys’ scalps correctly. The Hitachi ETG-4000 system automatically detects whether the contact adequately measures emerging photons for each channel.

### Data analysis

The analysis of fNIRS data in the present study was similar to that reported by Nakato et al. [26]. Before data analysis, we monitored the video recording of the boys’ behavior to evaluate good trials for analysis. We removed a trial from the analysis if either the accumulative looking time within the trial did not reach 5 s or if movement artifacts were detected by the analysis of sharp changes in the time-series of the raw fNIRS data.

The raw data on oxy-Hb, deoxy-Hb, and total-Hb concentrations from each channel were digitally band-pass-filtered at 0.02–1.0 Hz to remove noise caused by heartbeat pulsations or any longitudinal signal drifts. The mean concentration of each channel within a subject was then calculated by averaging data across the trials in a time-series with a 0.1 s resolution.

Based on mean concentrations in the time-series, we calculated the Z-scores for oxy-, deoxy-, and total-Hb in the familiar face and the unfamiliar face conditions for each channel within a subject [7, 20, 24–27]. The Z-score at each time-point indicates the deviation of hemodynamic response from the baseline during the presentation of faces. The mean concentration of the Z-score at 3 s before the trial was used as a baseline. The Z-scores were calculated separately for oxy-Hb, deoxy-Hb, and total-Hb in the familiar face and unfamiliar face conditions.

The Z-scores obtained from the 12 channels within the left and right temporal areas were averaged across trials for every subject in order to increase the signal-to-noise ratio. Although raw NIRS data consist of relative values that cannot be averaged directly across subjects or channels, normalized data including Z-scores can be averaged regardless of the unit. This calculation of the Z-score is a reliable analysis for changes in Hb concentration in the brain regions under the sensors since the analysis is independent of the differential path length factor (DPF). On the basis of these methodological advantages for fNIRS data, we applied the same analysis of the Z-score in the present study as in previous fNIRS studies with children [7, 20, 24–27].

**Fig. 3:**
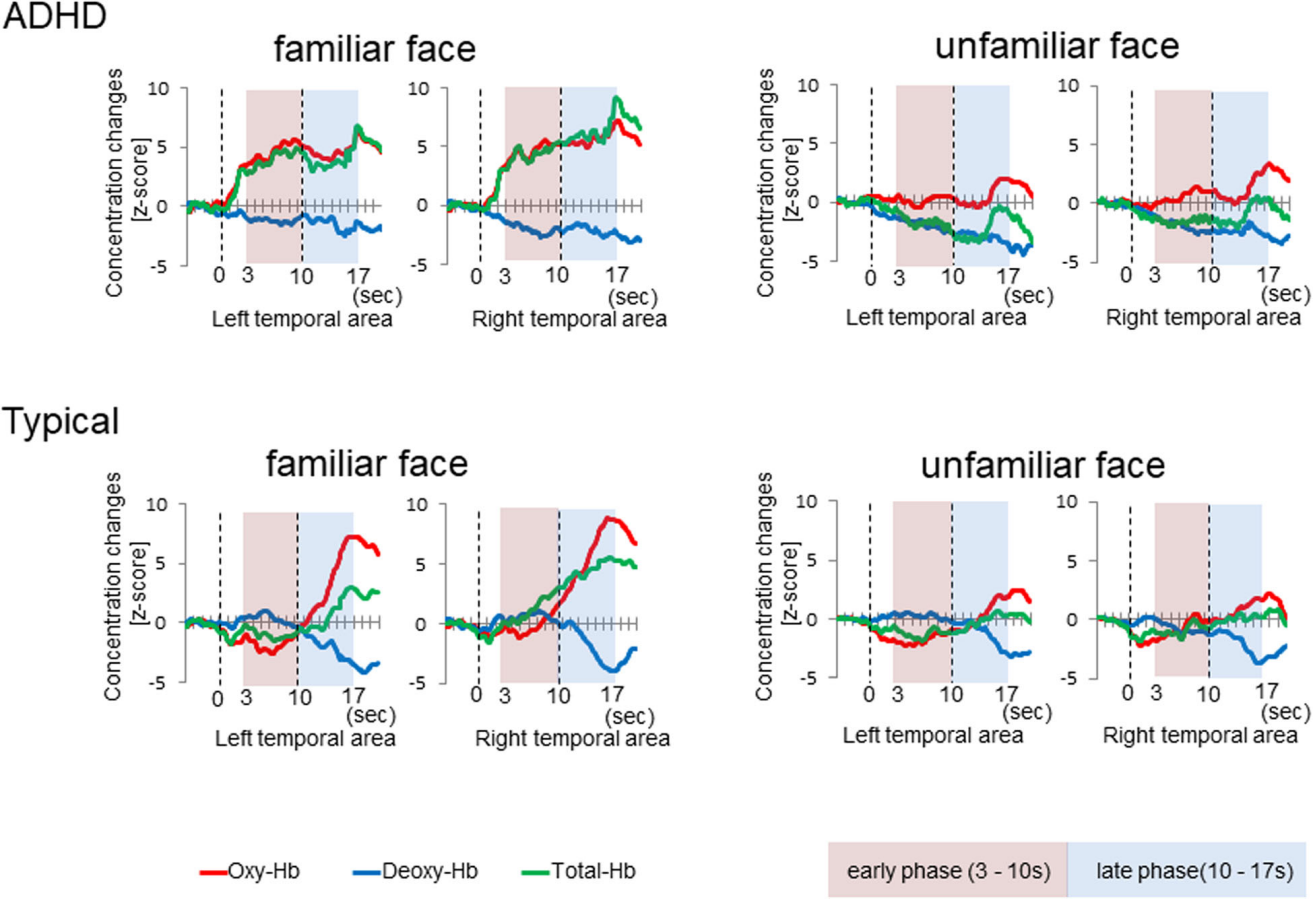
The time-course of the average change in oxy-, deoxy-, and total-Hb concentrations The time-course of the average change in oxy-Hb, deoxy-Hb, and total-Hb concentrations in the boys during the familiar face and unfamiliar face conditions. Zero on the horizontal axis represents the beginning of the test period and 10 represents the end of the test period. The areas in red and blue represent the zones for statistical analysis, i.e., the early phase (3–10 s) of the test period and late phase (10–17 s) after the test period

Consistent with our previous studies using fNIRS [7, 20, 24–27], we found a response peaking a few seconds after the stimulus onset. The hemodynamic response of oxy-Hb in the familiar face condition increased gradually from approximately 3 s after stimulus onset and reached a plateau approximately 10 s after stimulus onset in ADHD boys (see Fig. 3). In contrast, the hemodynamic response of oxy-Hb in the familiar face condition increased gradually from approximately 10 s after stimulus onset in TD boys (see Fig. 3). Therefore, we performed statistical analyses against the mean Z-scores for the 3–10 s (the early phase) and 10–17 s (the late phase) durations of the test period. A two-tailed one-sample *t*-test against a chance level of 0 (baseline) was conducted for the mean Z-score during the early phase and late phase in both temporal areas.

## Results

### Available trials for statistical analysis

Data from the 23 boys who looked at faces over more than three trials in both the familiar face and unfamiliar face conditions were obtained. Available trials were averaged across trials for each condition (familiar face, mean 5.06, SD 0.89; unfamiliar face, mean 4.97, SD 0.95). The mean number of removed trials for the familiar face condition was 0.33 (SD 0.74) while that for the unfamiliar face condition was 0.30 (SD 0.64). There was no significant difference between the numbers of available trials under each condition.

### Neuroimaging data

On the basis of the wavelengths of the Hitachi ETG-4000 system (695 nm and 830 nm), estimations of oxy-Hb concentration have been reported to better reflect changes in regional cerebral blood flow than deoxy- or total-Hb concentrations [29].

**Fig. 4:**
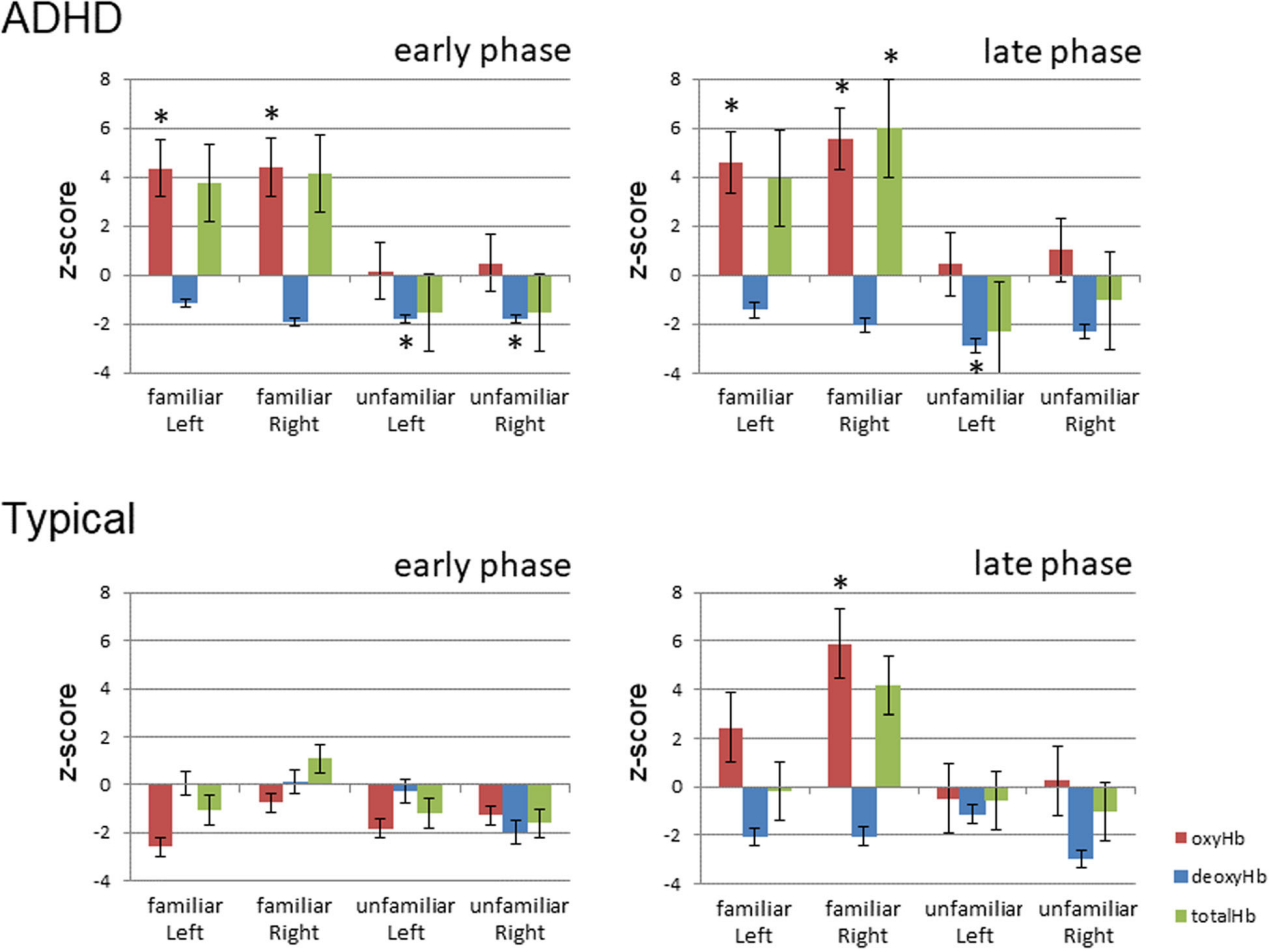
The mean Z-score during the early phase and the late phase. The mean Z-score during the early phase and the late phase in the left and right temporal cortices. The vertical bar in the graphs represents one standard error (SE). The left graph shows the data during the early phase and the right graphs shows the late phase. In the familiar face condition with ADHD, oxy-Hb concentrations (red bars) in the bilateral temporal cortex were significantly greater both in the early and late phase than the chance level of 0. In the TD boys, oxy-Hb concentrations of the right temporal cortex during the late phase were increased in the familiar face condition (red bars) (* *p* < .05)

The time-course of the average change in oxy-, deoxy-, and total-Hb concentrations in the boys during the presentation of the familiar face and unfamiliar face conditions is shown in Fig. 3. To examine the possibility that the activity for both the familiar and unfamiliar face stimuli were greater or smaller than the baseline, we conducted a two-tailed one-sample *t*-test with the mean Z-scores for oxy- deoxy-, and total-Hb concentrations for the 3–10 s (the early phase) and 10–17 s (the late phase) durations of the test period in each familiar and unfamiliar face condition (Fig. 4).

### The Z-scores for oxy-Hb concentrations

In the familiar face condition, the Z-scores for oxy-Hb concentrations in the ADHD boys were significantly increased in both temporal cortices in the early phase: right temporal cortex; *t* (8) = 3.80, *p* = 0.005; left temporal cortex; *t* (8) = 2.79, *p* = 0.023. They also showed a significant increase in both temporal cortices in the late phase: right temporal cortex; *t* (8) = 5.27, *p* = 0.0007; left temporal cortex; *t* (8) = 2.53, *p* = 0.035. However, in the unfamiliar face condition, there was no significant change in the concentration of oxy-Hb in the ADHD boys in either the early or late phase. TD boys showed no significant change in the early phase; however, in the late phase, they showed significant increases in the familiar face condition in the right temporal cortex: *t* (13) = 3.12, *p* = 0.008. Three-way ANOVA (2 × 2 × 2: group x condition x hemisphere) showed the only main effect in the condition (group *p* = 0.27; condition *p* = 0.011; hemisphere *p* = 0.087).

### The Z-scores for deoxy-Hb concentrations

The Z-scores for deoxy-Hb concentrations of the ADHD boys showed no significant change in the familiar face condition. However, in the unfamiliar face condition, the Z-scores of the ADHD boys were significantly decreased in both the right and left temporal cortices in the early phase: right temporal cortex; *t* (8) = 2.47, *p* = 0.039; left temporal cortex; *t* (8) = 4.97, *p* = 0.001. In the late phase, they showed significant decreases in the left temporal cortex: *t* (8) = 2.42, *p* = 0.041. TD boys showed no significant change in the concentration of deoxy-Hb in either condition in either the early or late phase.

### The Z-scores for total-Hb concentrations

The Z-scores for the total-Hb concentrations in the ADHD boys showed no significant change in either condition in the early phase. However, in the late phase, they showed significant increases in the right temporal cortex (*t* [8] = 2.67, *p* = 0.021), but only in the familiar face condition. TD boys showed no significant change in the concentration of the total-Hb in either condition in both the early and late phases.

## Discussion

The current study compared the neural correlates of viewing familiar and unfamiliar facial images in ADHD and TD boys using fNIRS. To measure the neural activities of facial recognition, the fNIRS probes were set on the bilateral temporal regions, including on the STS, which plays an essential role in processing faces. These measuring procedures were the same as in previous studies [7, 20, 23–27]. We calculated the Z-scores for the 12 channels within the temporal area from the raw data in the familiar and unfamiliar face conditions.

fNIRS monitors blood volume and oxygenation in the brain. In this study, the hemodynamic response, as indicated by oxy-Hb, increased gradually during the test period in both groups and was found to occur predominantly in the right hemisphere in TD boys. Neuroimaging studies have reported that the major aspects of facial processing arise predominantly in the right hemisphere [30, 31]. Therefore, these findings suggest that the hemodynamic response in this study indicates facial recognition in ADHD and TD boys. In the familiar face condition, Z-scores of oxy-Hb concentrations were significantly increased in the early and in the late phase for ADHD boys. In contrast, for TD boys, significant changes were only found in the late phase. In the unfamiliar face condition, we found no significant changes in either group of boys. This indicates that the hemodynamic response in ADHD boys to familiar faces may be earlier than TD boys. However, this research finding may not be related to the major ADHD symptoms of hyperactivity, impulsivity, and inattention. Gobbini et al. documented that familiar faces are recognized by processes that operate outside the focus of attention and without visual awareness [32]. In addition, numerous studies have reported slower reaction time variability among individuals with ADHD compare to TD individuals [33, 34]. Another possibility is that the ADHD boys did not show adaptation for familiar faces. Generally, repeated facial presentation induces neural adaptation [35].

Hemodynamic responses to familiar faces were found to occur predominantly in the right temporal area in TD boys. In contrast, ADHD boys showed significantly increased concentration of oxy-Hb to familiar faces in both the right and left temporal areas compared with the baseline. The TD boys showed right hemispheric dominance in processing faces, which is consistent with many other neuroimaging studies [30, 31]. Whereas the right hemispheric dominance in TD boys was indicated and successfully monitored by fNIRS, ADHD boys did not show right hemispheric lateralization to familiar faces. This finding is consistent with previous studies [6, 7]. Ichikawa et al. showed that the brain activity stimulated by seeing a happy face is significantly increased in both the right and left temporal areas compared with the baseline in ADHD boys.

Aside from the major symptoms of ADHD, boys with ADHD have been documented to have social cognitive impairments with impaired facial recognition and expression perception [4, 6, 7]. Additionally, in terms of basic facial processing, ADHD boys show a reduced face inversion effect on P1 latency compared to TD children using ERPs [8]. The inversion effect is an important index of facial expertise. These findings suggest that individuals with ADHD may have impairments of facial perception and recognition, which is consistent with the results of our study. There are a few limitations in the present study. First, we had difficulties with the measurement of non-medicated ADHD boys because of motion artifacts by their hyperactivity. It is better to remove the effect of medication, however, the effect of medication in facial perception and recognition is unclear in children with ADHD. Some previous studies showed that medication did not affect the recognition of facial expression in ADHD children [36] or slightly improved it [6], and there were some studies that did not examine the effect of medication [37, 38]. The second limitation was the small number of participants. After removing participants whose fNIRS data failed the motion criteria, only 69.7% of the subjects who were recruited remained in the study. Finally, although this is the first study to evaluate the neural correlates of familiar and unfamiliar facial recognition in children with ADHD, we recruited only boys in the current study. Future studies should examine a larger number of participants, recruit both genders, and consider the effect of medication.

In conclusion, we have demonstrated that differential brain activity occurs between ADHD and TD boys in response to the sight of a familiar face. During familiar face processing, ADHD children showed significant activity in both the early and late phase, while TD children showed significant activity only in the late phase. Additionally, the dominance of the right temporal areas for facial processing was found only in TD children during the familiar face presentation. These findings of atypical patterns of brain activity in ADHD boys may be related to social cognitive impairments in ADHD. We believe that these insights make a number of important additions to the literature and would be of considerable interest to researchers of biopsychosocial medicine.

## Data Availability

The datasets analyzed in this study are available from the corresponding author upon reasonable request.

## References

[1] Wolraich M, Brown L, Brown RT, DuPaul G, Subcommittee on Attention-Deficit/Hyperactivity Disorder, Steering Committee on Quality Improvement and Management (2011). ADHD: Clinical Practice Guideline for the Diagnosis, Evaluation, and Treatment of Attention-Deficit/Hyperactivity Disorder in Children and Adolescents. Pediatrics.

[2] American Psychiatric Association., American Psychiatric Association. DSM-5 Task Force. Diagnostic and statistical manual of mental disorders : DSM-5.

[3] Landau S, Moore LA (1991). Social skills deficits in children with attention-deficit hyperactivity disorder. School Psych Rev.

[4] Uekermann J, Kraemer M, Abdel-Hamid M, Schimmelmann BG, Hebebrand J, Daum I (2010). Social cognition in attention-deficit hyperactivity disorder (ADHD). Neurosci Biobehav Rev.

[5] Dawson G, Webb SJ, McPartland J (2005). Understanding the nature of face processing impairment in autism: insights from behavioral and electrophysiological studies. Dev Neuropsychol.

[6] Williams LM, Hermens DF, Palmer D, Kohn M, Clarke S, Keage H (2008). Misinterpreting emotional expressions in attention-deficit/hyperactivity disorder: evidence for a neural marker and stimulant effects. Biol Psychiatry.

[7] Ichikawa H, Nakato E, Kanazawa S, Shimamura K, Sakuta Y, Sakuta R (2014). Hemodynamic response of children with attention-deficit and hyperactive disorder (ADHD) to emotional facial expressions. Neuropsychologia..

[8] Tye C, Mercure E, Ashwood KL, Azadi B, Asherson P, Johnson MH (2013). Neurophysiological responses to faces and gaze direction differentiate children with ASD, ADHD and ASD + ADHD. Dev Cogn Neurosci Elsevier.

[9] Sinzig J, Morsch D, Lehmkuhl G (2008). Do hyperactivity, impulsivity and inattention have an impact on the ability of facial affect recognition in children with autism and ADHD?. Eur Child Adolesc Psychiatry.

[10] Johnston RA, Edmonds AJ (2009). Familiar and unfamiliar face recognition: a review. Memory..

[11] Itz ML, Schweinberger SR, Kaufmann JM (2016). Effects of Caricaturing in Shape or Color on Familiarity Decisions for Familiar and Unfamiliar Faces. PLoS One.

[12] Watanabe E, Yamashita Y, Maki A, Ito Y, Koizumi H (1996). Non-invasive functional mapping with multi-channel near infra-red spectroscopic topography in humans. Neurosci Lett Elsevier.

[13] Schroeter ML, Bücheler MM, Müller K, Uludağ K, Obrig H, Lohmann G (2004). Towards a standard analysis for functional near-infrared imaging. Neuroimage..

[14] Fukuda M (2012). Near-infrared spectroscopy in psychiatry. Brain Nerve.

[15] Ichikawa H, Kitazono J, Nagata K, Manda A, Shimamura K, Sakuta R (2014). Novel method to classify hemodynamic response obtained using MULTI-channel fNIRS measurements into two groups: exploring the combinations of channels. Front Hum Neurosci.

[16] Monden Y, Dan I, Nagashima M, Dan H, Uga M, Ikeda T (2015). Individual classification of ADHD children by right prefrontal hemodynamic responses during a go/no-go task as assessed by fNIRS. NeuroImage Clin.

[17] Araki A, Ikegami M, Okayama A, Matsumoto N, Takahashi S, Azuma H (2015). Improved prefrontal activity in AD/HD children treated with atomoxetine: a NIRS study. Brain Dev.

[18] Nagashima M, Monden Y, Dan I, Dan H, Mizutani T, Tsuzuki D (2014). Neuropharmacological effect of atomoxetine on attention network in children with attention deficit hyperactivity disorder during oddball paradigms as assessed using functional near-infrared spectroscopy. Neurophotonics..

[19] Monden Y, Dan H, Nagashima M, Dan I, Tsuzuki D, Kyutoku Y (2012). Right prefrontal activation as a neuro-functional biomarker for monitoring acute effects of methylphenidate in ADHD children: an fNIRS study. NeuroImage Clin.

[20] Inoue T, Sakuta Y, Shimamura K, Ichikawa H, Kobayashi M, Otani R (2015). Differences in the Pattern of Hemodynamic Response to Self-Face and Stranger-Face Images in Adolescents with Anorexia Nervosa: A Near-Infrared Spectroscopic Study. PLoS One.

[21] Ehlis A-C, Bähne CG, Jacob CP, Herrmann MJ, Fallgatter AJ (2008). Reduced lateral prefrontal activation in adult patients with attention-deficit/hyperactivity disorder (ADHD) during a working memory task: a functional near-infrared spectroscopy (fNIRS) study. J Psychiatr Res.

[22] Allison P (2000). McCarthy. Social perception from visual cues: role of the STS region. Trends Cogn Sci.

[23] Lloyd-Fox S, Blasi A, Everdell N, Elwell CE, Johnson MH (2011). Selective cortical mapping of biological motion processing in young infants. J Cogn Neurosci.

[24] Otsuka Y, Nakato E, Kanazawa S, Yamaguchi MK, Watanabe S, Kakigi R (2007). Neural activation to upright and inverted faces in infants measured by near infrared spectroscopy. Neuroimage..

[25] Kobayashi M, Otsuka Y, Kanazawa S, Yamaguchi MK, Kakigi R (2012). Size-invariant representation of face in infant brain: an fNIRS-adaptation study. Neuroreport..

[26] Nakato E, Otsuka Y, Kanazawa S, Yamaguchi MK, Watanabe S, Kakigi R (2009). When do infants differentiate profile face from frontal face? A near-infrared spectroscopic study. Hum Brain Mapp.

[27] Nakato E, Otsuka Y, Kanazawa S, Yamaguchi MK, Kakigi R (2011). Distinct differences in the pattern of hemodynamic response to happy and angry facial expressions in infants--a near-infrared spectroscopic study. Neuroimage..

[28] Klem GH, Lüders HO, Jasper HH, Elger C (1999). The ten-twenty electrode system of the international federation. The International Federation of Clinical Neurophysiology. Electroencephalogr Clin Neurophysiol Suppl.

[29] Yamashita Y, Maki A, Koizumi H (2001). Wavelength dependence of the precision of noninvasive optical measurement of oxy-, deoxy-, and total-hemoglobin concentration. Med Phys.

[30] Sergent J, Ohta S, MacDonald B (1992). Functional neuroanatomy of face and object processing. A positron emission tomography study. Brain.

[31] Tsuchiya N, Kawasaki H, Oya H, Howard MA, Adolphs R (2008). Decoding face information in time, frequency and space from direct intracranial recordings of the human brain. PLoS One.

[32] Gobbini MI, Gors JD, Halchenko YO, Rogers C, Guntupalli JS, Hughes H (2013). Prioritized Detection of Personally Familiar Faces. PLoS One.

[33] Kofler MJ, Rapport MD, Sarver DE, Raiker JS, Orban SA, Friedman LM (2013). Reaction time variability in ADHD: a meta-analytic review of 319 studies. Clin Psychol Rev.

[34] Tamm L, Narad ME, Antonini TN, O’Brien KM, Hawk LW, Epstein JN (2012). Reaction time variability in ADHD: a review. Neurotherapeutics..

[35] Kobayashi M, Otsuka Y, Kanazawa S, Yamaguchi MK, Kakigi R (2014). The processing of faces across non-rigid facial transformation develops at 7 month of age: a fNIRS-adaptation study. BMC Neurosci.

[36] Schwenck C, Schneider T, Schreckenbach J, Zenglein Y, Gensthaler A, Taurines R (2013). Emotion recognition in children and adolescents with attention-deficit/hyperactivity disorder (ADHD). ADHD Atten Deficit Hyperact Disord.

[37] Miller M, Hanford RB, Fassbender C, Duke M, Schweitzer JB (2011). Affect recognition in adults with ADHD. J Atten Disord.

[38] Da Fonseca D, Seguier V, Santos A, Poinso F, Deruelle C (2009). Emotion understanding in children with ADHD. Child Psychiatry Hum Dev.

